# Extremely Rare Pulmonary Metastases of Skin Basal Cell Carcinoma: Report of Two Cases with Clinicopathological Features

**DOI:** 10.3390/biomedicines11020283

**Published:** 2023-01-19

**Authors:** Małgorzata Edyta Wojtyś, Kajetan Kiełbowski, Michał Kunc, Seweryn Adam Skrzyniarz, Piotr Lisowski, Rafał Becht, Paulina Żukowska, Konrad Ptaszyński, Janusz Wójcik

**Affiliations:** 1 Department of Thoracic Surgery and Transplantation, Pomeranian Medical University in Szczecin, Alfreda Sokołowskiego 11, 70-891 Szczecin, Poland; 2 Students’ Scientific Circle of the Department of Thoracic Surgery and Transplantation, Pomeranian Medical University in Szczecin, 70-891 Szczecin, Poland; 3 Department of Pathomorphology, Medical University of Gdansk, 80-214 Gdańsk, Poland; 4 Dietrich-Bonhoeffer-Klinikum, 17036 Neubrandenburg, Germany; 5 Department of Clinical Oncology, Chemotherapy and Cancer Immunotherapy, Pomeranian Medical University in Szczecin, Unii Lubelskiej 1, 71-252 Szczecin, Poland; 6 Department of Pathology, Faculty of Medicine, Collegium Medicum, University of Warmia and Mazury in Olsztyn, 10-561 Olsztyn, Poland

**Keywords:** basal cell carcinoma, metastasizing basal cell carcinoma, pulmonary metastasizing basal cell carcinoma, pulmonary metastasis

## Abstract

Basal cell carcinoma (BCC) is the most frequent human skin cancer, but metastasizing BCC (MBCC) is extremely rare, developing in approximately 0.0028% to 0.55% of BCC patients. Herein, we report two cases of pulmonary MBCC. The first one developed in a 72-year-old male who underwent surgical resection due to multiple recurrences and adjuvant radiotherapy. Immunohistochemistry showed that neoplastic cells expressed Ber-EP4, CK5/6, p63, EMA (focally), BCL-2, and CD10, but were negative for CK7, CK20, S100, estrogen and progesterone receptors, and TTF-1. The second case is a 64-year-old female treated with vismodegib. Clinicopathological features and differential diagnoses are described.

## 1. Introduction

Basal cell carcinoma (BCC) is the most frequent human skin cancer and is characterized by slow growth and local destruction. The lifetime risk of BCC occurrence in Caucasians is considered to be 30%. The development of BCC is significantly associated with ultraviolet radiation, and the tumor is often located on sun-exposed skin [1]. In contrast to the high incidence of BCC, metastasizing BCC (MBCC) is extremely rare, occurring in 0.0028% to 0.55% of BCC patients. Male sex, primary location in the head and neck region, and treatment resistance are some factors associated with increased risk of MBCC [2]. The aim of this report is to raise awareness of the occurrence of MBCC by describing two patients with pulmonary MBCC.

## 2. Case 1

**Figure 1:**
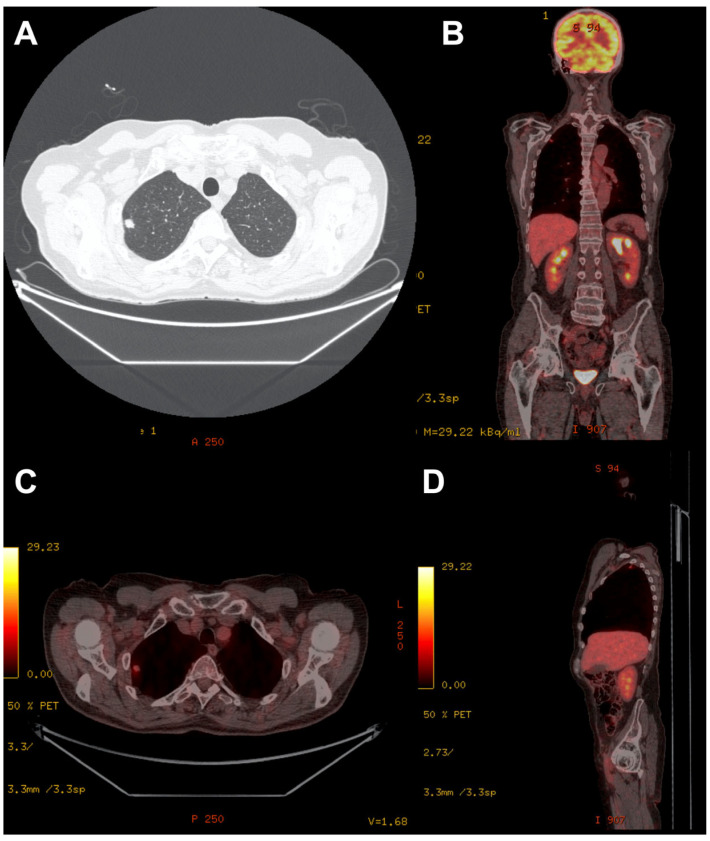
(**A**) Chest CT with a nodule in the right lung. (**B**). PET-CT coronal view. (**C**). PET-CT axial view. (**D**). PET-CT sagittal view. An 11 mm nodule is present in the second segment of the right lung, with a standardized uptake value of 2.45.

**Figure 2:**
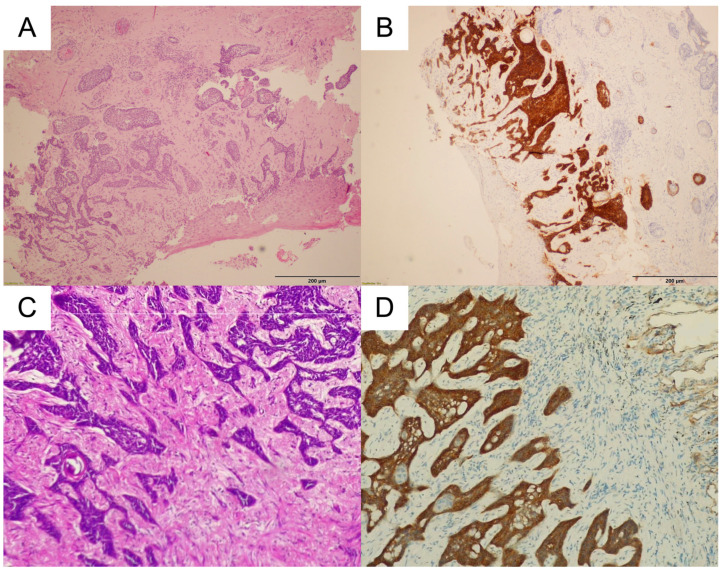
Histopathology of the basal cell carcinoma in patient 1. (**A**). Routine H&E staining of the recurrent BCC in the skin with ulceration and some scar formation (100×). (**B**). Staining with BerEP4 antibody showed an intense cytoplasmic reaction of the skin tumor. (**C**). Routine H&E staining of the metastatic BCC in the lung (200×). (**D**). Staining with BerEP4 antibody showed an intense cytoplasmic reaction of the metastatic tumor.

A 72-year-old male with a history of prostatectomy due to prostate cancer (2010) and resection of BCC on the skin of the face due to multiple recurrences with adjuvant radiotherapy (2014) was admitted to the Department of Pulmonology in May 2018 due to the discovery of a tumor in the right lung. Chest CT confirmed an 11 mm nodule in the second segment of the right lung, probably of post-metastatic character. However, bronchofiberoscopy showed no evident signs of neoplastic changes. As the etiology of the mass remained unclear, PET-CT was performed, revealing a standardized uptake value of 2.45 for the lesion in the second segment of the right lung (Figure 1). This result suggested a neoplastic background. It was decided that the patient needed to be treated surgically and was transferred to the Department of Thoracic Surgery and Transplantation in August of 2018. The thorax was entered through the fifth intercostal space. In the second segment of the right lung, a mass approximately 1.5 cm in diameter was palpated. It was approached by wedge resection, after which a specimen was sent for intraoperative histopathological examination. The intraoperative diagnosis was pulmonary metastasis of BCC. After 5 days of hospitalization, the patient was discharged home with instructions to return to the clinic for a general check-up and oncological consultation. According to the final histopathological examination, the resected pulmonary tumor was composed of nests of basaloid cells with distinctive peripheral palisading embedded in the fibrotic stroma, with a vague retraction artifact. Areas of pseudoglandular architecture were noted. Immunohistochemistry demonstrated that the neoplastic cells expressed Ber-EP4, CK5/6, p63, EMA (focally), BCL-2, and CD10, but were negative for CK7, CK20, S100, estrogen and progesterone receptors, and TTF-1. The microscopic picture was suggestive of metastasis of previously diagnosed cutaneous BCC; thus, the primary skin tumor specimen was retrieved for comparison. The histopathology of the primary skin lesion was reported to be nodular BCC. The subsequent recurrences showed ulceration and histological features of nodular BCC, with scarring and focally infiltrative growth. The morphology of the primary and metastatic tumors was similar and an immunohistochemical study with the antibody Ber-EP4 was positive, so the final diagnosis of pulmonary metastasis of cutaneous BCC was established (Figure 2). No intravascular tumor cells were found in the skin tumor sections. The patient is now under oncological control and has reported no signs of relapse.

## 3. Case 2

**Figure 3:**
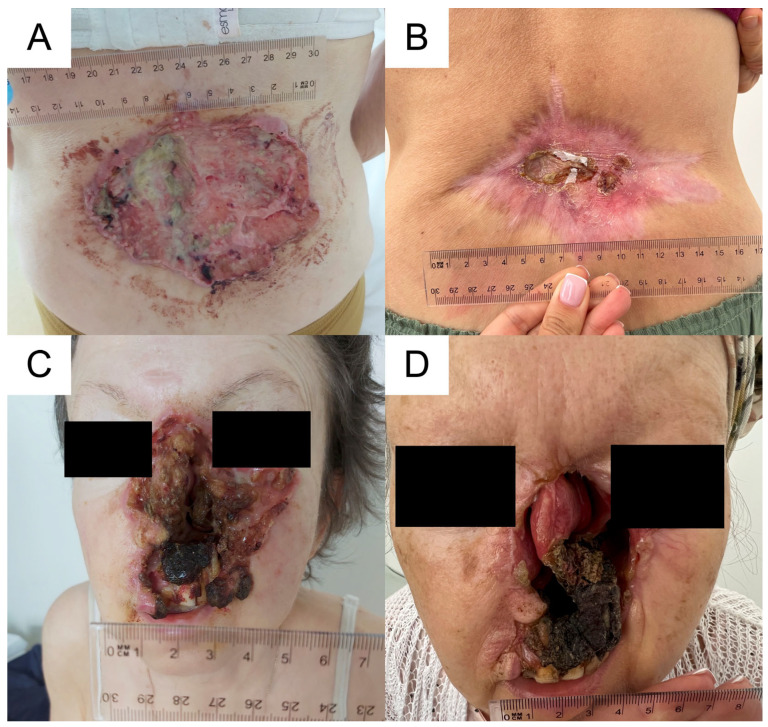
Face and lumbar ulcerations from BCC. (**A**). Before the vismodegib treatment, the primary lumbar lesion measured 16 cm × 11 cm. (**B**). After 1 year, the ulceration decreased to 1.5 cm with a 14 cm × 10 cm scar. (**C**). The primary face lesion measured 8 × 6 cm prior to treatment. (**D**). One year after treatment, the face lesion had decreased to 7 cm × 4 cm.

**Figure 4:**
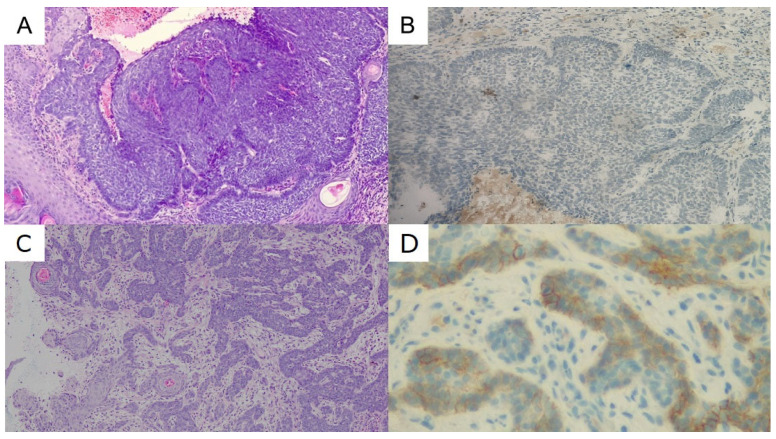
Histopathology of the basal cell carcinoma (BCC) in patient 2. (**A**). Routine H&E staining of the primary nodular BCC with ulceration on the face (100×). (**B**). Staining with Ber-EP4 antibody showed no positive reaction from the face lesion (100×). (**C**). H&E staining of the primary skin lesion of the lumbar region with infiltrative growth pattern (100×). (**D**). Staining with BerEP4 antibody showed a membranous reaction from the tumor cells of the lumbar region (400×).

**Figure 5:**
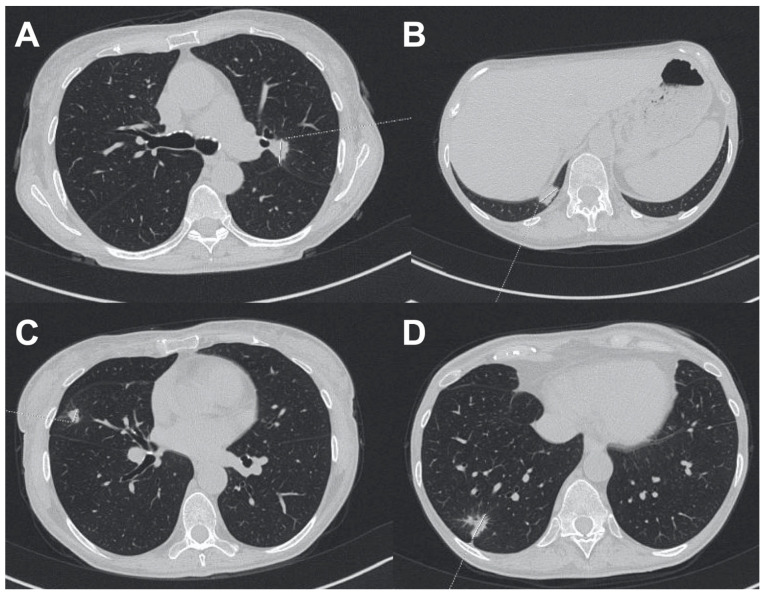
(**A**–**D**)**.** CT of patient 2, with multiple pulmonary nodules found in the right and left lungs.

A 64-year-old female patient, a retired family doctor without other concomitant diseases, experienced disseminated and long-lasting BCC, with the nose and lumbar region being the sites of the primary lesions. The socioeconomic status of the patient was relatively high. The patient was aware of the disease but likely suffered from adaptive and anxiety disorders, which is why she did not start the diagnostics and treatment at once. The patient denied a psychiatric and psychological consultation. Furthermore, the patient was reluctant to talk about the beginning of the disease and the reason why she did not consult a dermatologist or surgeon. The daughter of the patient explained more regarding the beginning and progression of the BCC on her face. The skin lesions had been observed for the previous 10 years, with progression in the last 4 years. There was extensive infiltration with destruction of the skin in the lumbar region and ulceration of the face (nasal cavities and upper lip) with palate fistula (Figure 3). The histopathological diagnosis of BCC was made in August 2021 (Figure 4). Histology of the lesion on the face showed features of nodular BCC with large nests of basaloid cells. Peripheral palisading and a retraction artifact were observed around some of the nests. Staining with Ber-EP4 was negative. In contrast, the lumbar lesion showed an infiltrating growth pattern and weakly positive Ber-EP4 membranous staining. In September 2021, the patient was admitted to the Department of Thoracic Surgery and Transplantation for further evaluation of an abnormal chest x-ray. Chest CT confirmed a paravertebral lesion in the 10th segment of the right lung (20 mm) and in the 9th segment of the same lung, infiltrating the parietal pleura (21 mm; Figure 5). Histology of the lung biopsy showed no viable tissue and was not diagnostic. The patient was disqualified from operation and discharged from the hospital. The stage of the disease precluded both surgical treatment and radiotherapy. Therefore, in October 2021, targeted systemic therapy was started. The patient received vismodegib, an inhibitor of the Hedgehog pathway, at the standard dose. After 9 months of treatment, local stabilization was achieved in the area of the face and the neoplastic ulceration on the skin in the lumbar region was significantly reduced. On the control CT, a visible reduction in metastatic changes within the lungs was observed. This led us to the conclusion that these were also of BCC origin. Currently, the patient is continuing the therapy with very good tolerance. Anemia was observed initially, most likely as a result of chronic inflammation. We are seeing an improvement in red blood cell counts. The patient does not currently require red blood cell transfusions. We have been observing significant improvement in the quality and comfort of her life since the beginning of systemic treatment, especially due to reduced ulceration, pain, unpleasant smell, and discharge from the lumbar lesion. 

## 4. Discussion

BCC comprises approximately 70%–80% of all skin cancers, and its incidence is increasing by 1%–3% annually. The vast majority of patients develop BCC after 40 years of age [3]. There are various histological subtypes of BCC, but the nodular and superficial types are the most common [4]. In case 1, primary ulceration from BCC was located on the face (right cheek) and measured 20 mm. In case 2, there were primary lumbar and face ulcerations from BCC. The histopathological features of the primary lesion in case 1 were reported as nodular, and those in case 2 as nodular (face) and infiltrative (lumbar region).

BCC develops from epidermal stem cells, hair follicle stem cells, or keratinocytes of the interfollicular epidermis [5,6]. In addition to sun exposure and fair skin, immunosuppression, genodermatoses (albinism, xeroderma pigmentosum), and ionizing radiation are risk factors for BCC development [7]. Surgery is considered a first-line treatment for primary BCC, with a 2%–8% 5-year postoperative risk of recurrence [8]. However, these rates change depending on the surgical technique and increase with time (10-year risk: 4.4–12.2%) [9]. 

MBCC is not frequently reported, and approximately 350–400 cases have been described in the English-language literature. Lymph nodes, lungs, and bones are the most common sites of BCC metastases [10]. Many authors have identified true MBCC cases based on the Lattes and Kessler classification developed in 1951 (excluding direct cancer spread, histopathological confirmation of primary BCC and metastasis, and a primary tumor located on the skin and not on a mucous membrane), and the presented patients fulfill those criteria [11,12]. Previous studies have reported a median survival of patients with MBCC of 8 to 14 months. However, according to the Kaplan–Meier survival curve reported by McCusker et al., the median survival for patients with distant metastases is 24 months and the 1-year survival rate is 58.6% [12]. Various factors have been associated with high metastatic potential [13]. Large primary tumors, invasion of blood vessels or perineural spaces, location in the head and neck region, multiple recurring primary tumors, condition after radiotherapy, immunosuppression, and fair skin, as well as male sex, have been described as risk factors for developing MBCC [14]. No perineural or lymphovascular invasion was found in either cases 1 or 2.

Lung manifestation of MBCC usually involves multiple nodules due to hematogenous transmission [15]. In our series, patient 1 developed a solitary pulmonary nodule, whereas multiple nodules were observed in patient 2. The differential diagnosis of pulmonary nodules found on chest CT is broad and includes neoplastic, infectious, or congenital origins, among others. Nevertheless, in patients with a history of cancer, metastatic nodules should always be considered a possibility. Treatment of MBCC usually involves surgery, radiotherapy, and targeted therapy. The pathogenesis of BCC strongly involves abnormal activation of the Hedgehog (Hh) pathway, which controls tissue repair and cell proliferation [16]. The Hh inhibitor vismodegib is the first FDA-approved drug to be used in patients with MBCC [17]. Successful adjuvant treatment with vismodegib has been reported in patients with pulmonary MBCC [18]. Locally advanced BCCs have demonstrated a complete response to vismodegib in 21% of patients and an assessed response rate of 43%, whereas the assessed response rate in patients with MBCC was 30% [19]. Mochel et al. described 22 cases of MBCC, including 11 pulmonary metastases. Primary locations included the face and neck, shoulder, chest, and abdomen. All patients with pulmonary MBCC underwent surgery [20]. The differential diagnosis of basaloid malignancies in the lungs includes basaloid squamous cell carcinoma, small cell carcinoma, adenoid cystic carcinoma, NUT carcinoma, poorly differentiated carcinomas (either of squamous or glandular differentiation), and metastases [21]. Immunohistochemistry is crucial and always necessary; the usual immunohistochemistry panel dedicated to pulmonary tumors, consisting of p40 or p63, TTF-1, and neuroendocrine markers, together with microscopic findings, is sufficient to establish the diagnosis. Distinguishing between metastases of BCC and adenocarcinoma, NUT carcinoma, and small cell carcinoma is usually straightforward. In doubtful cases, immunohistochemistry aids the diagnostic process because BCC is negative for TTF-1, NUT, and neuroendocrine markers. However, potential diagnostic pitfalls emerge when it comes to differentiating MBCC from primary basaloid squamous cell carcinoma (bSCC) of the lung. The latter is a very aggressive variant of lung cancer in which microscopic features overlap with BCC [22]; it forms nodular and trabecular structures with peripheral palisading, exhibits high mitotic activity, and is composed of small cells with scant cytoplasm. Both BCC and basaloid squamous cell carcinoma express squamous markers (p63, p40) and high-molecular-weight cytokeratins (e.g., CK5/6, CK14). In patients with a history of BCC, immunohistochemistry for CD10 or Ber-EP4 should be performed because, in contrast to basaloid squamous cell carcinoma, these markers are expressed by BCC [23,24]. In cases 1 (face) and 2 (lumbar region), the primary skin lesions stained positive with BerEP4 antibody.

In every case of suspected BCC metastasis, confrontation with the primary specimen is necessary, as it could have been misdiagnosed. Occasionally, lesions mimicking metastasis of BCC may be metastases of other cutaneous malignancies of appendageal or epidermal origin, such as high-grade trichoblastic carcinoma arising in trichoblastoma [25], pilomatrix carcinoma [26], and bSCC [27]. Johansson et al. reported a case of complex histology in a patient with pulmonary MBCC. The patient was considered to have metastatic bSCC, but histopathological reexamination with BerEP4, CK14, and CK17 immunohistochemical staining was performed to finally confirm MBCC. In addition, the patient did not tolerate treatment with vismodegib and was treated with cemiplimab, a PD-1 inhibitor, resulting in clinical improvement after 3 months of therapy [28].

## 5. Conclusions

In conclusion, we reported two cases of pulmonary MBCC manifesting as solitary and multiple pulmonary nodules on chest CT. Metastases, although rare, may develop years after resection of the primary lesion, as in patient 1, or result from long-lasting and disseminated disease, as in patient 2. Awareness of the risks of metastasis from skin BCC needs to be higher. The patient in the second case did not react to extensive lesions in the face and lumbar areas, despite being a doctor. Pulmonary MBCC should always be included in the differential diagnosis if the medical history involves BCC, especially in the head and neck region. Identification of pulmonary MBCC is also not straightforward in histopathological examination due to its similarities to basaloid squamous cell carcinoma. Early diagnosis of metastatic disease in BCC enables systemic treatment with new targeted drugs, such as the Hh pathway inhibitor vismodegib, or with immunotherapy by PD-1 blockade with cemiplimab in patients with progression or intolerance to a Hh pathway inhibitor. Modern systemic treatment of MBCC gives good clinical results, but most importantly, improves the quality of life for patients [29,30,31,32,33].

## Data Availability

Not applicable.
